# Investigation into the combustion kinetics and spontaneous ignition of sweet sorghum as energy resource

**DOI:** 10.1186/s40643-022-00536-0

**Published:** 2022-04-28

**Authors:** Numan Luthfi, Tappei Ohkoshi, Yutaka Tamaru, Takashi Fukushima, Kenji Takisawa

**Affiliations:** 1 Department of Environmental Science and Technology, Graduate School of Bioresources, Mie University, 1577 Kurimamachiyacho, Tsu, Mie 514-8507 Japan; 2 Staff Service, 85 Kandaneribeicho, Chiyoda-ku, Tokyo, 101-0022 Japan; 3 Department of Life Sciences, Graduate School of Bioresources, Mie University, 1577 Kurimamachiyacho, Tsu, Mie 514-8507 Japan

**Keywords:** Sweet sorghum, Thermogravimetric analysis, Frank-Kamenetskii theory, Combustion kinetics, Spontaneous ignition

## Abstract

This study investigated the combustion kinetics and spontaneous ignition of sweet sorghum using thermogravimetric analysis and the Frank-Kamenetskii theory. The aim was to determine the proper operating conditions for a direct combustion reactor and predict the safe ambient temperature limits for given silo designs. Oxidative heating rates of 2, 5, and 10 °C/min were set up. Graphical observation shows that combustion was composed of two different stages representing the overlapping processes of pyrolysis and char oxidation, at 131–336 °C and 336–475 °C, respectively. Samples were found to ignite at 215 °C and were extinguished at 433 °C. Different heating rates shifted combustion characteristics to higher temperatures and increased reactivity for ignition and combustion indices up to 12 and 10 times higher. The Friedman method determined the apparent activation energies representing the combustion reaction by 132.91 kJ/mol. Regarding spontaneous ignition, the temperature safe limits were predicted to be 83–84 °C and 84–87 °C for cylindrical and box silos with diameter and height of 15 and 10 m, respectively. Calculations of silos were designed within the limits of certain dimension ratios.

**Graphical Abstract:**

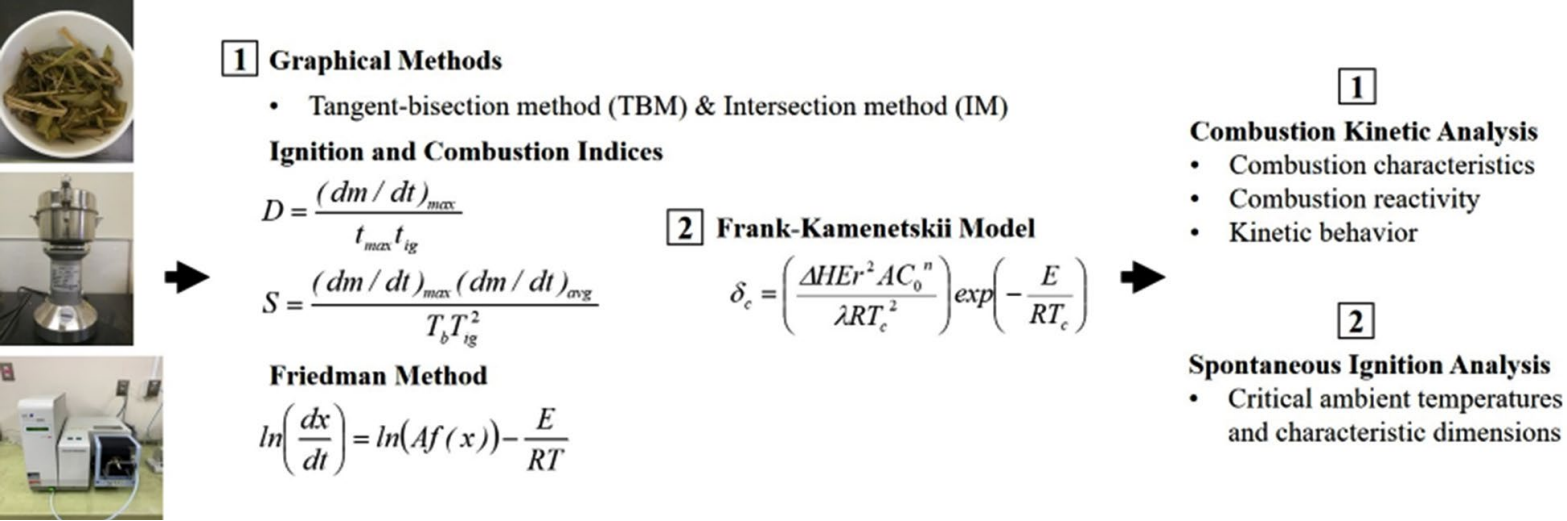

## Introduction

The priority of utilizing biomass has attracted much global attention to address environmental problems, such as global warming, due to the excessive use of fossil fuels. Those concerns are reflected in IRENA’s 1.5 °C scenarios on climate change, where bioenergy represents 25% of the total estimated primary supply or equals 153 EJ by 2050 (IRENA 2021). Hence, the exploration of various potential biomass for the power generation industry will be intensively carried out to meet the needs.

For years, studies of combustion kinetic and spontaneous ignition have been proposed to understand the essential characteristics of biomass during utilization and storage before being applied in large-scale power industries. Direct combustion has become an efficient technology for converting biomass into energy and has been widely used in power plants with steam turbine systems. Thermal behavior is crucial information to explain the ignition and burnout of fire which influences boiler operation, energy efficiency, and emissions (Cao et al. 2017). Therefore, understanding combustion kinetic can help better knowledge of design and optimization in biomass combustion systems. Meanwhile, spontaneous ignition may arise from the exothermic process due to the susceptibility of stockpiled materials to self-heating. The physical, biological, and chemical heating mechanisms are addressed to contribute to self-heating events (Sheng and Yao 2022). There were many reports of fire incidents during storage. For instance, a fire started from a large biomass pile of 500 tons at Advanced Agro-Power Plant in Thailand on 12 March 2017 due to accumulated heat. For that reason, the knowledge of spontaneous ignition can give a fundamental evaluation and mitigation of fire hazards in biomass storage.

Thermogravimetric analysis (TGA) has received immense attention for studying the combustion kinetics of biomass. Many studies have used TGA to profile its characteristic temperatures (i.e., ignition temperature, burnout temperature, and peak temperature(s)), kinetic parameters (i.e., activation energy and pre-exponential factor), and reactivity parameters (i.e., ignition index and combustion index) in an oxidative environment. The traced thermal degradation reveals the response of combustion reaction to various operating conditions, such as biomass composition, heating rate, and others. The experimental study using olive residues was done by Magalhaes et al. (2017). The characteristic temperatures were graphically identified from the TG and DTG curves at 240–350, 340–700, 240–245, and 531–703 °C for two combustion reaction stages, ignition, and burnout, respectively. The apparent activation energies, which are 44.2–47.7 and 9.1–13.4 kJ/mol, were presented by assuming reaction mechanisms and calculating them using the model-fitting method of Coats–Redfern.

Meanwhile, spontaneous ignition can be predicted in a biomass stockpile by considering its combustion kinetic parameters and applying the mathematical theory of Frank-Kamenetskii. The theory expresses that the ignition arises based on solid heat conduction in specific geometric shapes. Boonmee and Pongsamana (2017) performed a laboratory-scale experiment using bagasse. A cylindrical- or rectangular-shaped stockpile with any radius or length stored with asymptotic heights below 10, 7.8, and 6 m is considered safe from fire hazards at ambient temperatures of 40, 45, and 50 °C.

The current study focused on a comprehensive investigation of combustion kinetics and spontaneous ignition using sweet sorghum. Sorghum was chosen to represent the primary marginal crop, which has many superior characteristics compared to other crops, such as growing in a short period of about 4–6 months and being able to withstand an environment with little water and high soil salinity (Xie et al. 2018). TGA was performed at different heating rates and in an oxidative environment to assess its response to reaction stages, characteristic temperatures, kinetic parameters, and reactivity parameters. Subsequently, the spontaneous ignition was predicted by substituting the existing kinetic parameters into the steady-state solution of the Frank-Kamenetskii equation. The prediction was presented regarding the relationship between ambient temperature and safe silo size.

## Materials and methods

### Characteristics and preparation of sweet sorghum samples

Sweet sorghum was collected locally from farm areas around Mie University, Japan. Sorghum was appropriately washed to eliminate dirt and was air-dried (± 20 °C) for several days to reduce moisture content and inhibit decay. Dried sorghum samples were ground using a high-speed blender, YKB (AS ONE Corp.), with a rotation speed of approximately 28,000 rpm for 1 min. All samples were sieved to a size range of 250–500 μm. According to Wilen et al. (1996), the proximate and ultimate characteristics of sweet sorghum are summarized in Table 1.

**Table 1:** Proximate and ultimate characteristics of sweet sorghum (Wilen et al. 1996)

Proximate analysis	Value^db^ (%)	Method
Moisture content	7.04arb	DIN 51,718
Ash content	4.74	DIN 51,719
Volatile matter	77.20	DIN 51,720
Fixed carbon	18.06	–

Ultimate analysis	Value^db^ (%)	Method
Carbon	47.30	–
Hydrogen	5.80	–
Oxygen	41.67	By difference
Nitrogen	0.40	–
Sulphur	0.09	ASTM D 4239

### TGA experimental approach

A thermal analyzer, EXSTAR 6000 TG/DTA 6200 (Seiko Instruments Inc.), was used to conduct the thermogravimetry (TG), derivative thermogravimetry (DTG), and differential thermal analysis (DTA) experiments. Air was fed into the furnace from the pump and was regulated to 100 ml/min for each experiment. Samples with a mass of 6 mg were used in each experiment and were heated from ambient temperature to 550 °C at three different heating rates of 2, 5, and 10 °C/min. The experiment was conducted 2 times under the same conditions to verify the reproducibility. A small sample mass and low heating rate were applied to avoid the transport effect and enhance the resulting signal of slower kinetic events.

### Combustion kinetic analysis

#### Determination of reaction stages

**Fig. 1:**
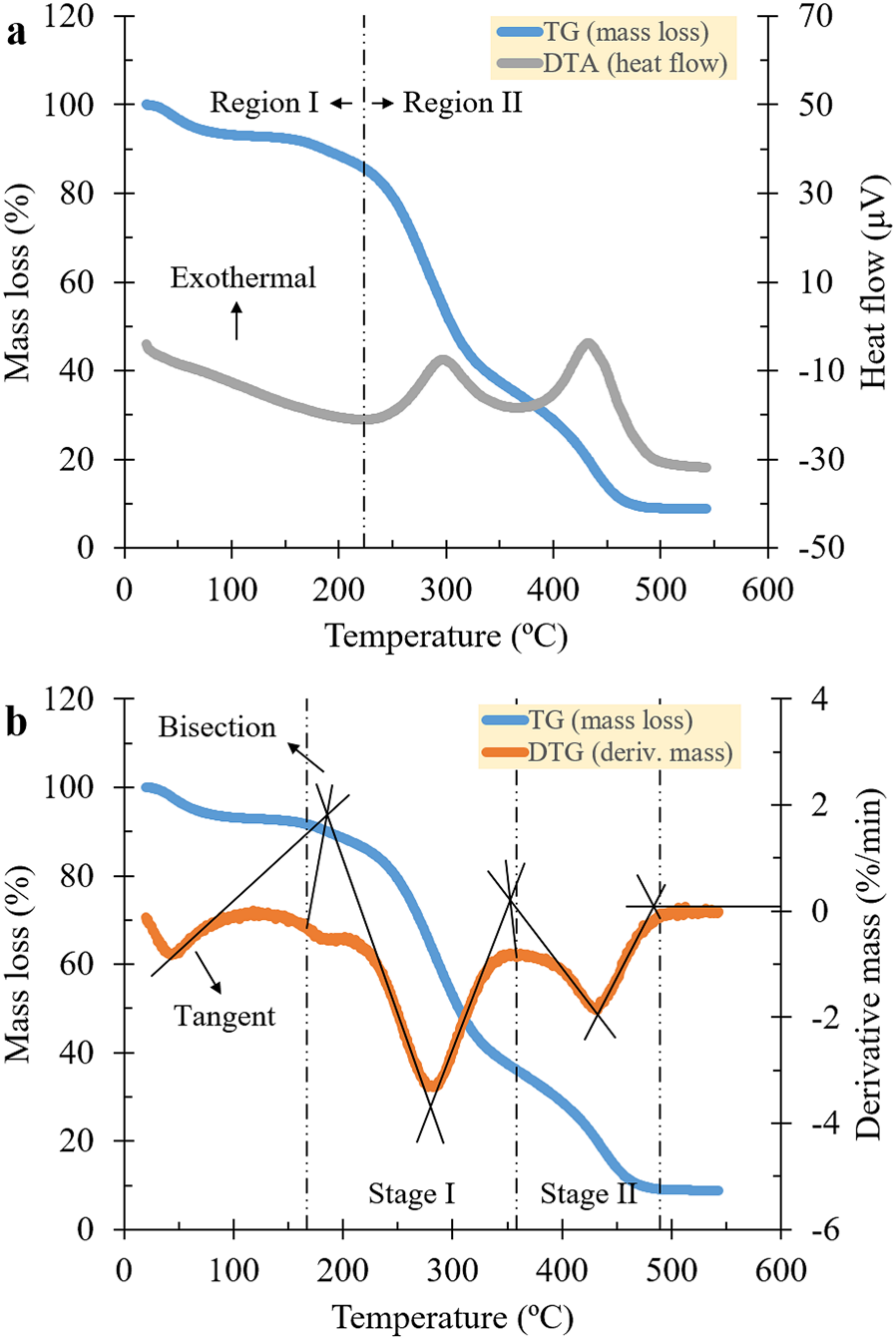
Schematic for determining **a** the combustion reaction region and **b** combustion reaction stages (e.g., at a heating rate of 5 ºC/min; 1st iteration sample)

The combustion reaction region (exothermic reaction) was qualitatively determined by observing the gain portion of the DTA curve (see Fig. 1a). Then, by following the method of Pickard et al. (2013) or tangent-bisection method (TBM), the reaction stages of combustion were identified on a DTG curve with the assumption that they are non-competing, first-order, and single-stage reactions obeying the Arrhenius law (see Fig. 1b). Tangent lines were drawn to the edges of the leading and trailing curves. Bisection lines were extended at each tangent intersection until they reached the DTG trace and labeled as the start or end of the reaction stages.

#### Determination of characteristic temperatures

**Fig. 2:**
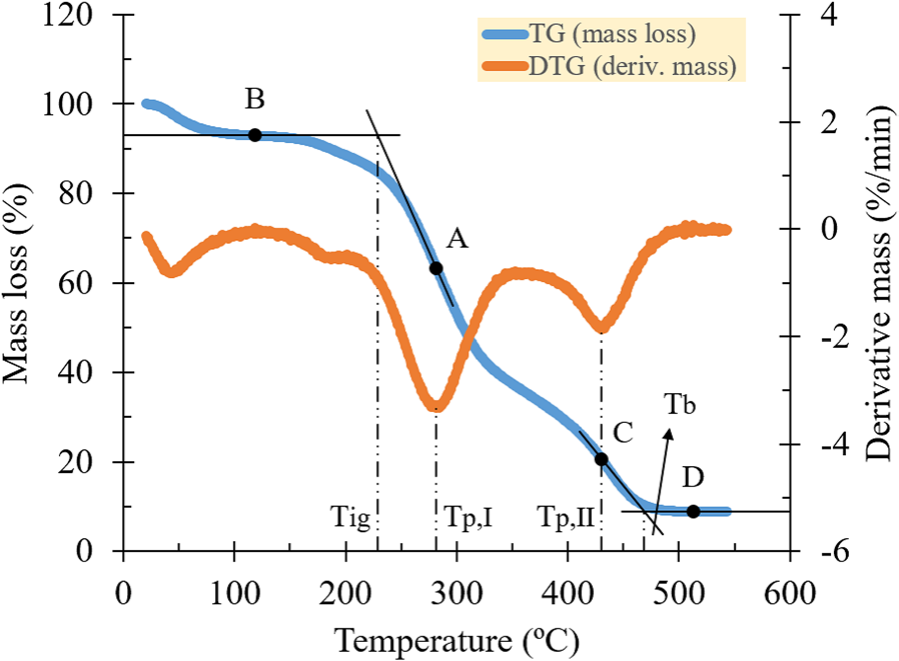
Schematic for determining the peak, ignition, and burnout temperatures (e.g., at a heating rate of 5 ºC/min; 1st iteration sample)

The characteristic temperatures were determined using a TG and DTG curve, based on the report of Lu and Chen (2015) (see Fig. 2). Peak temperatures were observed at the maximum mass loss rate of each reaction stage. Then, the intersection method (IM) was employed to observe the ignition and burnout temperatures. Horizontal lines were drawn at points B and D, where the TG curve became steady after the evaporation and combustion reactions were complete. Afterward, tangent lines were drawn at points A and C through the horizontal lines of points B and D, respectively. Points A and C are defined as cross points at which vertical lines from the DTG curve peaks of stages I and II cross the TG curve. The temperatures corresponding to the intersections of the tangent and horizontal lines were labeled as the ignition and burnout temperatures, respectively.

#### Determination of reactivity parameters

In support of the reactivity data, the ignition and combustion indices were calculated for all samples to provide a more accurate measure of the conversion over time and temperature. According to Fraga et al. (2020b), the ignition and combustion indices are defined in Eq. 1 and Eq. 2, respectively:

1 $$D = \frac{{\left( {dm/dt} \right)_{\max } }}{{t_{\max } t_{ig} }},$$

2 $$S = \frac{{\left( {dm/dt} \right)_{\max } \left( {dm/dt} \right)_{avg} }}{{T_{b} T_{ig}^{2} }},$$

where *D* and *S* are the ignition (in %/min^3^) and combustion indices (in %/min^2 ^°C^3^). *(dm/dt)*_*max*_ and *(dm/dt)*_*avg*_ are the maximum and average mass loss rates (in %/min), respectively. *T*_*ig*_ and *T*_*b*_ are the ignition and burnout temperatures (in °C). *t*_*ig*_ and *t*_*max*_ are the times corresponding to the ignition and maximum combustion rates (in min).

#### Determination of kinetic parameters

The Friedman method, which obeys the Arrhenius law, was used to estimate the kinetic data from a TG curve. Since the amount of airflow is much greater than the sample mass used, the oxidation process is assumed not to depend on the oxygen concentration. Therefore, the combustion reaction was modeled as first-order kinetics.

According to Yao et al. (2008) and Huang et al. (2016), the combustion of solid biomass is described by the following:

3 $$\frac{dx}{{dt}} = kf\left( x \right),$$

where *dx/dt* is the conversion rate (in 1/s), *k* is the reaction rate constant (in 1/s), and *f(x)* is the reaction function. In addition, *x* is the degree of conversion. The expression for *x* is defined as:

4 $$x = \frac{{m_{i} - m_{t} }}{{m_{i} - m_{f} }},$$

where *m*_*i*_, *m*_*t*_, and *m*_*f*_ are the initial mass of the sample, mass of the sample at a specified time, and final mass of the sample (in kg), respectively. Furthermore, the expression of *k* is defined by the Arrhenius equation:

5 $$k = Ae^{ - E/RT},$$

where *A* is the pre-exponential factor (in 1/s), *E* is the activation energy (in J/mol), *R* is the universal gas constant (in J/molK), and *T* is the temperature of the sample (in K).

Subsequently, substituting Eq. 5 into Eq. 3 gives the following equations:

6 $$\frac{dx}{{dt}} = Af\left( x \right)e^{ - E/RT}.$$

The model-free method of Friedman is defined in Eq. 7 by taking a logarithm on both sides of Eq. 6 without assuming the reaction model and the reaction function. The following equation is hence obtained:

7 $$\ln \left( {\frac{dx}{{dt}}} \right) = \ln \left( {Af\left( x \right)} \right) - \frac{E}{RT}.$$

By plotting the left-hand side of Eq. 7 against *1/T* at different heating rates followed by taking a linear regression, the activation energy (*E*) can be obtained from the slope. Meanwhile, the intercept may be used to determine the pre-exponential factor (*A*) if the reaction function is modeled beforehand (for first-order kinetics, it is *f(x)* = *(1-x)*).

### Spontaneous ignition analysis

The safe size of the stockpile and ambient temperature under critical condition were evaluated using the dimensionless equation of Frank-Kamenetskii. The equation is expressed by Eq. 8, which represents the ratio of chemical energy to thermal conduction (Boonmee and Pongsamana 2017; Fisher and Goetz 1993):

8 $$\delta_{c} = \frac{{\Delta HEr^{2} AC_{0}^{n} }}{{\lambda RT_{c}^{2} }}\exp \left( { - \frac{E}{{RT_{c} }}} \right),$$

where *δ*_*c*_ is the critical Damkohler number, and *T*_*c*_ is the critical ambient temperature (in K). *r* is the characteristic dimension of a pile with origin at the center (in m), which is half of the total depth (*r* = *1/2r*_*0*_) or equal to the total radius (*r* = *r*_*0*_). *ΔH* is the heat reaction (in J/kg), which is assumed to be equivalent to the calorific value (*Q*) multiplied by the conversion degree (*x*) at the ignition temperature (*ΔH* = *x*_*ig*_*Q*). *C*_*0*_ and *λ* are the bulk density (in kg/m^3^) and thermal conductivity of the sample (in W/(mK)). n is the kinetic reaction order. *E* and *A* are the activation energy (in J/mol) and pre-exponential factor of reaction (in 1/s), respectively, which is obtained from kinetic analysis at the ignition temperature. *R* is the universal gas constant (in J/(molK)).

## Results and discussion

### Combustion reaction and characteristic temperatures of sweet sorghum

Based on the graphical analysis of DTA, sweet sorghum was thermally degraded in two distinct regions of exothermic and endothermic reactions due to evaporation (region I) and combustion (region II). As shown in Fig. 1a, the first gain in heat flow permits the identification of these two regions. The gain temperatures were identified at 226–223 °C for all heating rates. The negative-valued region asserts that sorghum samples absorbed heat to release surface and inherent moisture. In contrast, the positive-valued region corresponds to heat liberation due to lignocellulosic pyrolysis (i.e., dehydration and decarboxylation of hemicellulose, cellulose (> 400–450 °C), and lignin) and burning volatile and char (Rahib et al. 2019; Basu 2013). The heating rate affected the onset of heat release by lowering the gain temperature by 3 °C at higher heating rates and vice versa. It took place at approximately 226 °C for heating rates of 2 °C/min and 223 °C for rates of 5–10 °C/min. This is because the heat given at a low rate provided enough time for water to diffuse or even break their bonds from vessels and fibers completely (Penvern et al. 2020). The force holding might derive from hydrogen bonds among the water in vessels or between the water molecules and hydrophilic sites via functional groups, such as hydroxyls, phenolic, and carboxylic acids (Khare and Baruah 2014). The difference in the mass loss proves the presence of a water concentration gradient in the sample. The smaller the mass loss is, the more water remains. TG curve shows that the mass loss decreased from 82.51 to 86.80% at the transition point as the rate increased.

**Fig. 3:**
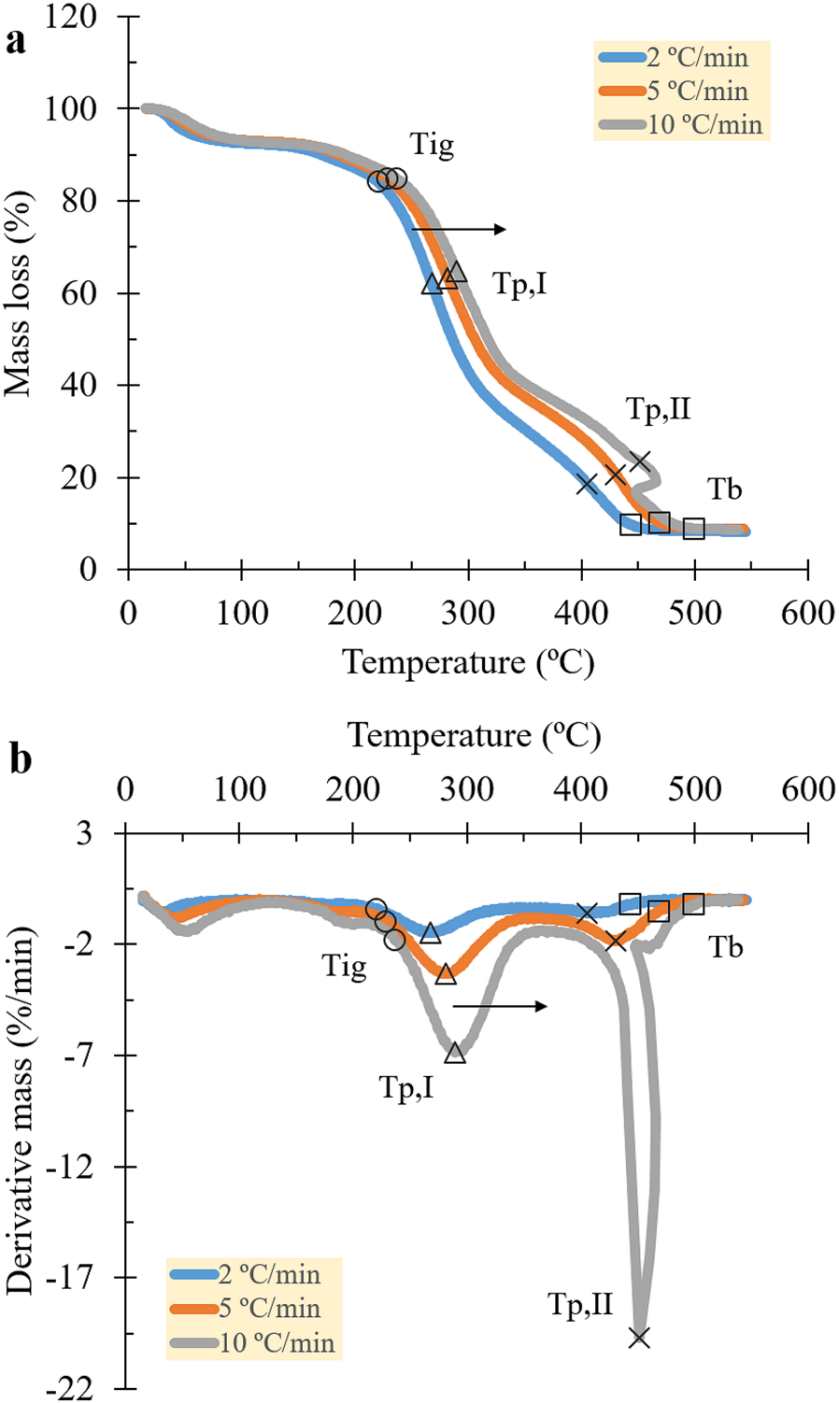
**a** TG and **b** DTG signals with plots of the characteristic temperatures for all heating rates (1st iteration sample)

The combustion region, profoundly, was identified as having multi-stage reactions, as depicted by the occurrence of slopes and peaks on the TG and DTG curves (see Fig. 3). The tangent-bisection and intersection methods were performed on the DTG curve to enhance confidence in quantifying the major reaction stages and characteristic temperatures (see Fig. 1b and Fig. 2). For each temperature parameter obtained (i.e., reaction temperatures and characteristic temperatures) from all heating rates, extrapolations were subsequently done at 0 °C/min to exhibit the material nature and eliminate the linear influence of the heating procedure. On the other hand, the remaining masses, showing the material nature, were determined by simply averaging the three corresponding heating rates due to intrinsic property (Yao et al. 2008). The obtained data are summarized in Table 2 and Table 3.

**Table 2:** Reaction temperatures and characteristic temperatures of the combustion reaction under different heating rates

Heating rate (°C/min)	Reaction temperature (°C)		Characteristic temperature (°C)			
	Stage I	Stage II	Tp,I	Tp,II	Tig	Tb
- (material nature)	131–336	336–475	264	405	215	433
2	143–348	348–475	268	412	218	444
5	168–362	362–486	281	435	229	467
10	197–391	391–487	292	452	236	495

**Table 3:** Mass changes including reaction durations of the combustion reaction under different heating rates

Heating rate (°C/min)	Remaining mass, % (standard deviation)		Mass loss, % (reaction duration, s)		Maximum mass-loss rate (%/min)	
	Stage I	Stage II	Stage I	Stage II	Tp,I	Tp,II
- (material nature)	91.01 (1.26)–34.14 (1.42)	34.14 (1.42)–9.70 (0.22)	56.87	24.44	–	–
2	91.95–32.55	32.55–9.80	59.40 (6057)	22.75 (3846)	1.42	0.66
5	91.50–35.28	35.28–9.45	56.22 (2265)	25.83 (1506)	3.38	1.86
10	89.59–34.59	34.59–9.84	55.00 (1110)	24.74 (585)	6.94	17.69

The robust methods divided the combustion reaction into two stages, which occurred at 131–336 and 336–475 °C for stages I and II, respectively. The first stage is attributed to the pyrolysis and volatile oxidation controlled by chemical reactions (Magalhaes et al. 2017). Slow pyrolysis (< 50 °C/min) produced volatiles and chars majorly through dehydration and decarboxylation of hemicellulose (127–235 °C), cellulose (227–350 °C), and lignin (127–527 °C) (Basu 2013; Carvalho et al. 2015), wherein the volatiles then diffused into the ambient air while the chars remained (Sami et al. 2001). In a sufficient proportion, the mixed gases and/or some solids were predicted to give off flames at 215 °C due to oxidation, when cellulose became the dominant mechanism (Lu and Chen 2015). The pyrolysis reaction consumed 56.87% of the sorghum mass, with the reactivity rate increasing sharply at 264 °C (*T*_*p,I*_). Meanwhile, the second stage corresponds to the char oxidation within the diffusion-controlled phase via mixed gas (Magalhaes et al. 2017). In situ oxidation of volatiles and chars might proceed in parallel due to overlapping. The transition from the burning of volatile to char can be notified by the change of flame into an ember (Rahib et al. 2019). After the volatile matter in the atmosphere was exhausted, oxygen-rich air diffused further into the interior of the char and burned out the rest to ashes (Sami et al. 2001). The ember was found to extinguish at 433 °C (*T*_*b*_). Overall, the oxidation reaction converted 24.44% of the sorghum mass with the highest reactivity at 405 °C (*T*_*p,II*_).

Following the preliminary identification, the appearance of the shoulder (less pronounced) and head peaks on the DTG curve at around 180–200 and 270–290 °C for all heating rates, respectively, indicates the pseudo-hemicellulose and pseudo-cellulose within the pyrolytic stage (see Fig. 3b). Both could also be marked at the first and second slopes of the TG curve, excluding the slope of water evaporation (see Fig. 3a). In contrast, the pseudo-lignin peak, which is believed to be indistinct due to its notoriously broad reaction, might fall around 320–420 °C according to the third TG slope. Meanwhile, the char oxidation reaction could be notified by the prominent DTG peak at 410–450 °C or the fourth TG slope. Similar locations of pseudo-lignocellulose were reported by Carvalho et al. (2015) on sweet sorghum in an inert atmosphere using the deconvolution method. Accordingly, Jayaraman et al. (2017) confirmed that pyrolysis and char oxidation occurred in similar ranges by investigating the evolution of typical gases from various biomass. A significant amount of water vapor (H_2_O), carbon monoxide (CO), hydrogen (H_2_), and aromatic compounds (i.e., methane (CH_4_), benzene (C_6_H_6_), and so on) was detected in the range of 150–400 °C, showing the typical gaseous products of dehydration, decarboxylation, and scission in pyrolysis reactions. In contrast, carbon dioxide (CO_2_) was released more in char oxidation between 400–750 °C along with a small release of CO, representing the partial oxidation in char.

Furthermore, increasing the heating rate affected the TG and DTG curves to shift to higher temperatures, marked by a significant delay of 10–50 °C for both temperature parameters as shown in Fig. 3 and Table 2. The significant linear influence is a consequence of the decrease in the time required for heat transfer to cross the biomass interior before the surface temperature increases (Elorf et al. 2021). A major deterrent to the higher heating rate is the low thermal conductivity of sorghum by 0.13 W/mK (Fennell and Boldor 2014). For this reason, even though the heating rate was high, the sorghum interior was heated at a considerably slower.

### Combustion reactivity of sweet sorghum

Changes in heating rate exhibit unique effects on the mass changes and reaction durations. Based on Fig. 3 and Table 3, the mass losses fluctuated during combustion with a tendency to decrease insignificantly (1–4%) as the heating rate increased. Stage I experienced a decrease in the mass loss by 59.40–55.00% at a heating rate of 2 to 10 °C/min. Meanwhile, the mass loss at stage II increased from 2 to 5 °C/min by 22.75–25.83% and decreased from 5 to 10 °C/min by 25.83–24.74%. Fluctuating results were also obtained in the report of Fraga et al. (2020a) and Jayaraman et al. (2017) using various biomasses and broader ranges of heating rates. Fraga et al. (2020a) deduced this to be a form of randomness. Additionally, the insignificance of the mass changes was expressed in small standard deviations (SD) of the remaining masses, namely 1.24 (1.38%; relative standard deviation (RSD)), 1.42 (4.16%), and 0.22% (2.27%) for the onset of stage I, transition point, and offset of stage II, respectively. This implies that the mass change was probably an intrinsic property and not affected by the heating rate (Yao et al. 2008). Conversely, the increase in heating rate alone significantly affected the reaction durations, denoted by the decrease from 6057 to 1110 s during stage I and from 3846 to 585 s during stage II. This implies that the combustion reaction proceeded faster at higher heating rates without any obvious change in the amount of mass converted.

The maximum mass-loss rates at the corresponding stages rose from 1.42 to 6.94%/min and from 0.66 to 17.69%/min as the heating rate increased, agreeing with the mass loss to reaction duration ratio (see Table 3). The values define the rate at which the non-condensed and condensed phases of biomass decompose to gases. As can be observed, stage II, which was dominated by char, had lower mass-loss rates than stage I due to the high energy bond of the carbon–carbon bond. Correspondingly, the susceptibility of sorghum characterized by a high fuel ratio (> 2.0) of volatile matter to fixed carbon (4.27) exerted influences on the results of the early stage (Lu et al. 2013; Wilen et al. 1996), such as low ignition temperature (200–300 °C) and higher maximum mass-loss rate during devolatilization (see Table 1) (Basu 2013). Comparing the rate magnitude of the two, stage II rose approximately ten times higher under 5–10 °C/min. In contrast, stage I increased merely twofold at the same level of heating rate. It may be caused by the ability of oxygen to overcome mass transfer resistance and diffuse at high heating rates (Islam et al. 2016). Unlike the devolatilization process which is only sensitive to the particle temperature, the reactivity of char oxidation can accelerate due to temperature elevation and oxidizer concentration (Li et al. 2016), which may affect the present findings in the similar way.

**Fig. 4:**
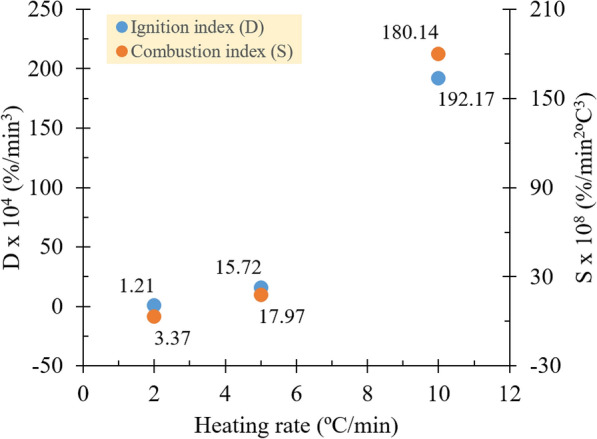
Effects of different heating rates on the ignition and combustion indices

Supporting the given trends in the maximum mass-loss rate, the change in heating rate shows a directly proportional relationship with the ignition and combustion indices. Both indices signify the decomposition over time and temperature within combustion stages. The ignition and combustion indices were obtained to increase from 1.21 × 10^4^ to 192.17 × 10^4^%/min^3^ and from 3.37 × 10^8^ to 180.14 × 10^8^%/min^2^°C^3^, respectively, under 2–10 °C/min heating rates (see Fig. 4). Substantially, the ignition index suggests that sorghum caught fire faster with magnitudes of approximately 13–12 times higher as the heating rate increased. This index is assigned by concerning the amount of volatile evolution within the first combustion stage or pyrolysis reaction. Meanwhile, the combustion index exhibits the activity of the substance consumption, starting when the sorghum caught fire until the chars burned out into ashes, signifying the predominance of oxidation. Following the same previous causal relationship, the entire combustion reaction proceeded rapidly by the increment of about 5- to 10-fold (Wnorowska et al. 2021; Fraga et al. 2020b).

### Kinetic behavior of sweet sorghum

**Fig. 5:**
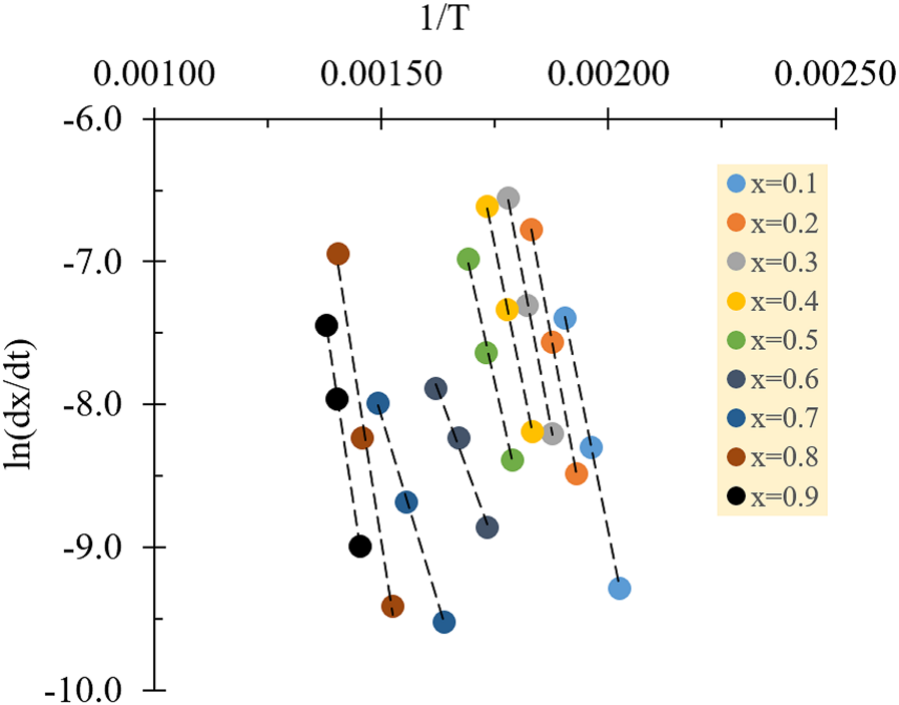
Linear plots of ln(dx/dt) against 1/T for various conversion degree in Friedman method representing the entire combustion reaction

The Friedman method was employed to evaluate the kinetic data of the overall combustion reaction by linearly fitting the term *ln(dx/dt)* to *1/T* in Eq. 7 for a series of conversion degrees at different heating rates. The conversion degree in the range of 0.1–0.9 was used in this study with segmentation of 0.1. Figure 5 shows that fitted lines were nearly parallel in three conversion ranges of 0.1–0.5, 0.6–0.7, and 0.8–0.9, denoting a single reaction or unification of several reactions (Yao et al. 2008). Averaging over the corresponding parallel ranges, then, will give meaningful activation energy. To avoid overestimation, the relative standard deviation (RSD) was determined to be no more than 10% (see Table 4). The correlation coefficient values demonstrated the coherence of kinetic parameters generated by the Friedman method. Fraga et al. 2020b stated that the first-order kinetic model can be considered to fit well if the coefficient is more than 0.90 (*R*^2^ > 0.90). Because the correlation coefficients of this study ranged from 0.9893 to 0.9999 for the whole conversion degree, these results indicate the adequacy of the kinetic parameters to represent good linear fitted plots and reaction mechanism (i.e., model and function).

**Table 4:** Activation energies, pre-exponential factors, and correlation coefficients (R^2^) for the entire combustion reaction using Friedman method

Conversion (mg/mg)	Friedman method			Conversion (mg/mg)	Friedman method		
	E (kJ/mol)	A (1/s)	*R*^2^		E_avg_ (kJ/mol)	SD	Relative SD
0.1	129.57	5.25 × 10^9^	0.9998	0.1–0.5	132.91	8.60	6.47
0.2	143.38	7.30 × 10^10^	0.9999	0.6–0.7	79.40	7.21	9.07
0.3	140.19	2.14 × 10^10^	0.9992	0.8–0.9	169.44	2.45	1.45
0.4	132.49	2.21 × 10^9^	0.9994				
0.5	118.92	6.00 × 10^7^	0.9961				
0.6	72.20	1.26 × 10^3^	0.9918				
0.7	86.61	6.30 × 10^3^	0.9994				
0.8	166.99	7.97 × 10^9^	0.9893				
0.9	171.89	1.41 × 10^10^	0.9993				

**Fig. 6:**
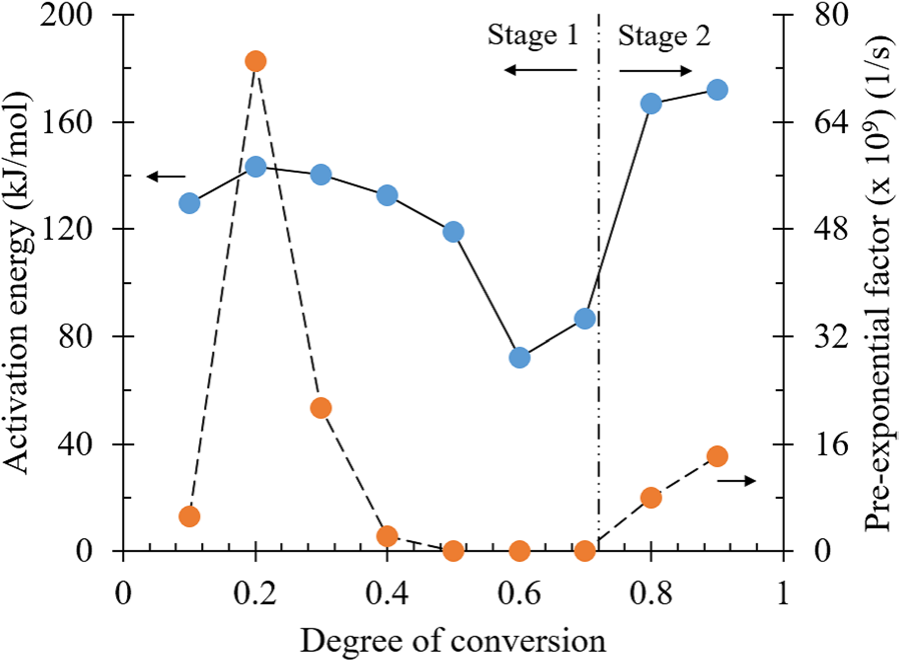
Apparent activation energy and pre-exponential factor as a function of conversion degree calculated by Friedman method

The distribution of activation energies and pre-exponential factors in the function of conversion degree is shown in Fig. 6. Converting the conversion degree of 0.1–0.5 to temperature range from 218 to 317 °C, which has the exact position in the applicable heating rates of 2, 5, and 10 °C/min, the unified reaction was allegedly suspected to be conjoint pyrolysis of hemicellulose and cellulose predominantly. The mean activation energy was found to be 132.91 kJ/mol, while the pre-exponential factor ranged from 5.25 × 10^9^ to 6.00 × 10^7^ s^−1^, indicating the frequency of molecular collision for the reaction to occur (see Table 4). Thus, the lower the activation energy is, the easier it is for a reaction to commence. Considering the same way to elucidate the data, lignin-dominated pyrolysis underwent in the second half of the conversion degree (0.6–0.7), settling in the temperature from 304 to 395 °C. The required activation energy decreased to 79.40 kJ/mol, presumably due to the least presence of hemicellulose and cellulose remaining at the high temperature. At a conversion degree of 0.8–0.9, equivalent to a temperature range from 386 to 453 °C, char oxidation was presumed to be the reaction to occur. The minimum energy for oxidation to react increased drastically to 169.44 kJ/mol as a consequence of the gradual deposition of carbon constituents. Additionally, both possible reactions were disclosed to have pre-exponential factors spreading from 1.26 × 10^3^ to 6.30 × 10^3^ and from 7.97 × 10^9^ to 1.41 × 10^10^ s^−1^, respectively.

It is important to note that satisfying activation energy is required to represent the entire reaction. According to Yao et al. (2008) and Luo et al. (2016), fractions below 0.5–0.7, showing parallel plots at the beginning, generally provide meaningful information instead of the whole process, especially for the case of self-heating and combustion. The activation energy, which encompasses the ignition process, determines the external energy to heat the fuel reactor since the subsequent reaction will become self-sustaining above this temperature, related to exothermic chain reactions. Therefore, the apparent activation energy of the combustion reaction in this study was 132.91 kJ/mol regardless of the heating rate.

### Spontaneous ignition of stockpiled sweet sorghum

The dimensionless parameter of Frank-Kamenetskii or critical Damkohler number (*δ*_*c*_), expressed by Eq. 8, was used to predict spontaneous ignition based on the energy equation at steady state. The thermal explosion model was derived by assuming that the Biot number is equivalent to infinity (*Bi* = *∞*). The number means that reactant temperature is distributed along the body in a parabolic manner, governed by internal conduction. Thus, the critical Damkohler number depends specifically on the geometry of reacting systems (Fisher and Goetz 1993).

Current work studied the relationship between critical ambient temperature and silo dimension in sorghum storage. Since feedstock is usually piled up in a non-geometric shape, the stockpile was assumed to comply with two common geometric shapes in the market: (1) a cylinder with dimensions of diameter (*d*) and height (*h*); and (2) a rectangular box with dimensions of height (*h*), length (*l*), and width (*w*). If the critical ambient temperature is set within a specific range, and the Damkohler number for a given geometry is specified, the response of the selected silo dimension to the critical temperature can be calculated. According to Fisher and Goetz (1993), Damkohler numbers for finite cylinder (heat loss one end) and rectangular box are shown in Eq. 9 and Eq. 10, respectively.

9 $$\delta_{c} = 2.0 + 0.195(d/h)^{2},$$

10 $$\delta_{c} = 0.825(1.067 + (h/l)^{2} + (h/w)^{2} ).$$

Regarding the calculations, the critical ambient temperature (*T*_*c*_) was set between 0–200 °C (273.15–473.15 K) to represent the relative reference that changed in the input (see Table 5). A series of diameter and height were generated as the selected dimensions of cylindrical and box silos, which are equivalent to twofold the characteristic dimension (*r*). Meanwhile, other storage dimensions were converted into ratios such as *d/h*, *h/l*, and *h/w*, so that Eq. 9 and Eq. 10 could be calculated mathematically and substituted into Eq. 8 (see Table 6). Concerning the rest parameter in Table 5, the thermal conductivity (*λ*) was cited from the work of Fennel and Boldor (2014), while the bulk density (*C*_*0*_) and calorific value (*Q*) were, respectively, experimented based on the method of Bhagwanrao and Singaravelu (2014) and the standard of adiabatic calorimeter (O.S.K 150, Ogawa Sampling Co., Japan). Upon extrapolating at 0 °C/min, the conversion degree of ignition (*x*_*ig*_) was multiplied by calorific value to determine the heat reaction (*ΔH*). Meanwhile, the activation energy (*E*) and pre-exponential factor (*A*) were fitted to the linear plots in Fig. 5, which satisfied the conversion degree of ignition of 0.102.

**Table 5:** Calculation parameters used in Frank-Kamenetskii model

Parameter	Value	Parameter	Value	Parameter	Value
*T*_*c*_ (K)	273.15–473.15	*ΔH* (J/kg)	17.79 × 10^5^	*E* (J/mol)	129.57 × 10^3^
*C*_*0*_ (kg/m^3^)	38.86	*Q* (J/kg)	173.91 × 10^5^	*A* (1/s)	5.25 × 10^9^
*λ* (W/mK)	0.13	*xig*	0.102	*R* (J/molK)	8.314

**Table 6:** Geometric shapes and dimension ratios used in Frank-Kamenetskii model

Geometric shape	Dimension ratio		Geometric shape	Dimension ratio		
	d/h	Note		h/l	h/w	Note
Cylinder	1/10 (0.1)	(h > d)	Box	1/2 (0.5)	1/2 (0.5)	(h < l,w) (l > w)
	1/3.3 (0.3)			1/4 (0.25)	1/2 (0.5)	
	1/2 (0.5)			1/8 (0.125)	1/2 (0.5)	
	1/1.4 (0.7)			1/4 (0.25)	1/4 (0.25)	
	1/1.1 (0.9)			1/8 (0.125)	1/4 (0.25)	
				1/16 (0.0625)	1/4 (0.25)	

**Fig. 7:**
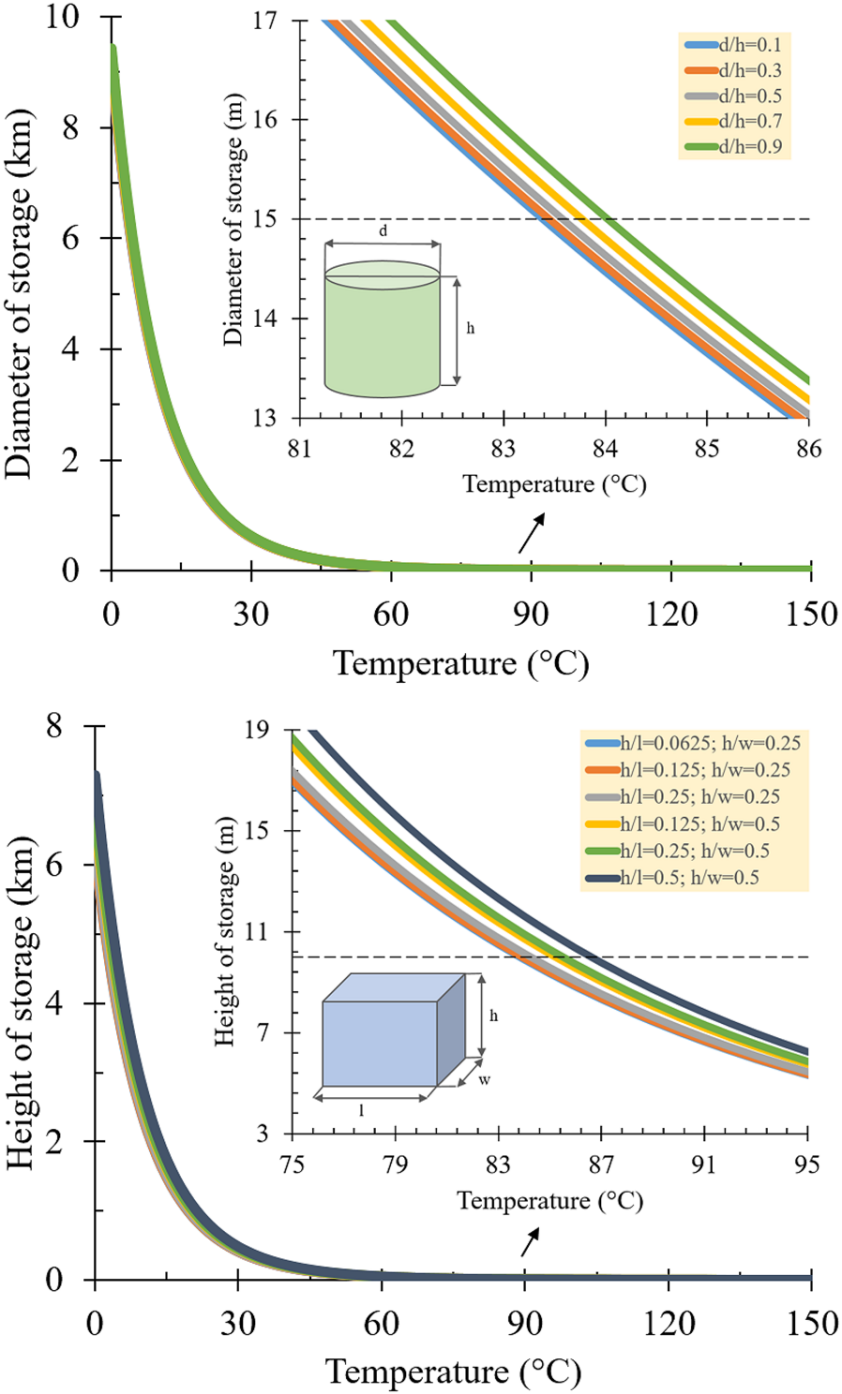
Relationship between the critical ambient temperature and selected silo dimension for **a** cylindrical and **b** rectangular-boxed shapes, including the enlarged curves to show the effect of different dimension ratios

Observing Fig. 7, the resulting curves suggest that at certain levels of temperature and silo size, the areas under the curves are appropriate for storing sorghum piles safely. The opposite meaning applies to the area above the curve. For both geometric shapes of cylinder and box, the curves exhibit vertical (va) and horizontal asymptotes (ha) around the coordinates *y*_va_ = 8 km; *x*_va_ = 60 °C and *y*_ha_ = 6 km; *x*_ha_ = 60 °C when the selected dimensions were increased or decreased to near infinity while keeping the respective ratios. The findings confirm the report of Murasawa et al. (2013) on storage of soy sauce residue and fishmeal, in which similar asymptotic responses were detected.

The market designs were employed to present enlarged curves around diameter and height of 15 and 10 m for both geometric shapes to study the spontaneous ignition of sorghum on a relevant scale (see Fig. 7a and Fig. 7b). These values were chosen because they are commonly used in large-scale industries and are decent to apply to several dimension ratios in the current study. Based on the cylindrical silo design (*d* = 15 m), the spontaneous ignition of sorghum will not occur at ambient temperature below 83–84 °C, within the increment of *d/h* ratio from 0.1 to 0.9. It is possible to notify that the shift due to various *d/h* ratios was only 1 °C. Similar findings were found in the box-shaped silo design (*h* = 10 m). The critical temperature showing a safe limit for spontaneous ignition ranged between 84–87 °C. The temperature shifted by 3 °C as the *h/l* and *h/w* ratios rose from 0.0625 to 0.5 and from 0.25 to 0.5, respectively. The combination of the two ratios is shown in Table 6. In addition to finding the critical temperatures for each design, although not significant, it can be seen that the smaller the resized dimension to the ratio is, the greater the ambient temperature for a fire to ignite is required, owing to the immense heat dissipation.

## Conclusions

The combustion kinetics and spontaneous ignition of sweet sorghum have been investigated successfully using TGA and the Frank-Kamenetskii theory. The findings are highly related to sorghum utilization as a fuel in a combustion reactor and to safety storage as a feedstock from fire hazards. Investigation shows that activation energy of 132.91 kJ/mol was required to undergo the combustion in a reactor, fitted well by the first-order model. The combustion of sweet sorghum occurred in the temperature range of 131–475 °C and comprised two different thermal stages, corresponding to pyrolysis and char oxidation. The flame was predicted to give off at 215 °C, and the sorghum was almost consumed completely at 433 °C. The effect of heating rate on reactivity suggests that reactor operation was preferably at 10 °C/min due to time effectiveness and no distinct mass change. The ignition and combustion indices were evidenced to rise to 12 and 10 times higher.

Regarding sorghum storage, the stockpile was assumed to comply with typical cylindrical and box silos in the large-scale industries, having diameter and height of 15 and 10 m, respectively. Based on *d/h* ratios of 0.1–0.9, sorghum in the designated cylindrical silo (*d* = 15 m) had critical temperature showing a safe limit between 83–84 °C. Meanwhile, the spontaneous ignition is not possible to occur at ambient temperature below 84–87 °C for the designated box silo (*d* = 10 m), within the increment of *h/l* and *h/w* combination ratios between 0.0625–0.5 and 0.25–0.5.

## Data Availability

All data analyzed during this study are included in this article.

## Abbreviations

TGA: Thermogravimetric analysis
TG: Thermogravimetry
DTG: Derivative thermogravimetry
DTA: Differential thermal analysis
TBM: Tangent-bisection method
IM: Intersection method
